# Combining heterogeneous data sources for accurate functional annotation of proteins

**DOI:** 10.1186/1471-2105-14-S3-S10

**Published:** 2013-02-28

**Authors:** Artem Sokolov, Christopher Funk, Kiley Graim, Karin Verspoor, Asa Ben-Hur

**Affiliations:** 1 Department of Biomolecular Engineering, University of California Santa Cruz, Santa Cruz, California 95064, USA; 2 Computational Bioscience Program, University of Colorado School of Medicine, Aurora, Colorado 80045, USA; 3 National ICT Australia, Victoria Research Lab, Melbourne 3010, Australia; 4 Department of Computer Science, Colorado State University, Fort Collins, Colorado 80523, USA

## Abstract

Combining heterogeneous sources of data is essential for accurate prediction of protein function. The task is complicated by the fact that while sequence-based features can be readily compared across species, most other data are species-specific. In this paper, we present a multi-view extension to GOstruct, a structured-output framework for function annotation of proteins. The extended framework can learn from disparate data sources, with each data source provided to the framework in the form of a kernel. Our empirical results demonstrate that the multi-view framework is able to utilize all available information, yielding better performance than sequence-based models trained across species and models trained from collections of data within a given species. This version of GOstruct participated in the recent Critical Assessment of Functional Annotations (CAFA) challenge; since then we have significantly improved the natural language processing component of the method, which now provides performance that is on par with that provided by sequence information. The GOstruct framework is available for download at http://strut.sourceforge.net.

## Introduction

The availability of a large variety of genomic data relevant to the task of protein function prediction poses a data integration challenge due to the heterogeneity of the data sources. While features based on sequence can be readily compared across species, most other data are species-specific: protein-protein interactions are probed experimentally in a given species, and the expression of a given gene measured in one set of experiments is difficult to compare meaningfully to expression measured in another species, under possibly different conditions.

In earlier work we have shown the power of modeling Gene Ontology (GO) term prediction as a hierarchical classification problem using a generalization of the binary SVM to structured output spaces, which allows us to directly predict the GO categories associated with a given protein [1]. Our results demonstrated that the GOstruct method achieves state-of-the-art performance on the Mousefunc competition dataset. In this work we generalize the GOstruct method to allow us to combine both species-specific data and cross-species data computed from sequence, using the framework of multi-view learning [2]. The multi-view learning approach learns a separate classifier for each set of features, and inference is performed jointly in order to predict a label. We demonstrate that the multi-view framework is able to utilize all available information, yielding better performance than sequence based models trained across species and models trained from collections of data within a given species. Preliminary results of the multi-view approach using a limited number of data sources were presented elsewhere [3]. This approach achieved state-of-the-art performance in the recent Critical Assessment of Functional Annotations (CAFA) challenge.

In addition to data that is commonly used in prediction of protein function, namely gene expression and protein-protein interactions (PPI), we report the successful use of large-scale data mined from the biomedical literature, and find that it provides a large boost in accuracy. Together with the text mining data, features based on sequence similarity and PPI account for most of the predictor performance.

We examined the tasks of predicting molecular function, biological process and cellular component in isolation. Our empirical results demonstrate that sequence-based data is more suited to inferring molecular function. Conversely, PPI-based classifiers do well in the other two tasks, outperforming predictors based on any other single source of data. Gene expression data and other sequence-based features provide a marginal increase in accuracy.

## Background

The Gene Ontology (GO) [4] is the current standard for annotating function. GO terms belong to three namespaces that describe a gene product's function: its function on the molecular level, the biological processes in which it participates, and its localization to a cellular component. Each namespace is structured as a hierarchy over its set of keywords, where keywords lower in the hierarchy provide greater specificity of description. Since a protein may have multiple functions in each GO namespace, the problem of protein function prediction can be formulated as hierarchical multi-label classification [5].

For a long time, the predominant approach to inferring GO function for newly sequenced proteins has been *transfer of annotation *[6], where annotations are transferred from proteins with known function on the basis of sequence or structural similarity. Many studies have shown the limitations and issues with this approach [7-10]. Nevertheless, a number of methods employ sequence and structural similarity to make functional annotation predictions with varying degrees of accuracy [11-15]. New schemes are still being proposed today, an example being the algorithm by Hamp, *et al*. that was used in the 2011 CAFA challenge [12].

The transfer-of-annotation approach operates like a nearest-neighbor classifier, and is unable to effectively deal with today's noisy high-throughput biological data. This has led to the recent development of machine learning approaches that typically address the problem as a set of binary classification problems: whether a protein should be associated with a given GO term (see e.g., [16]). The issue with breaking the problem up into a collection of binary classification problems is that the predictions made for individual GO terms will not necessarily be consistent with the constraint that if a term is predicted, all its ancestors in the hierarchy should be predicted as well. Therefore, some methods attempt to reconcile the predictions with the hierarchy to produce a set of consistent annotations e.g., using Bayesian networks or logistic regression [5,17,18]. Other methods employ inference algorithms on graphs to directly produce a hierarchical label [19,20]. But the common approach is to forgo the reconciliation step entirely, partly because the predominant approach to measuring prediction accuracy for this problem is on a "per GO term" basis [21]. In this case, the interpretation of potentially conflicting binary predictions is left up to the user.

The biomedical literature is a resource that has been previously explored for protein function prediction, including as the topic of a shared task ([22]). Several of the previous efforts in this area take advantage of machine learning (e.g. [23-25]), typically training a binary classifier for each GO term as in other related work, where the features employed in the models are derived from informative or discriminating words in text associated to a protein. While some of these approaches show promise, each paper also suggests that integration of external data sources would be useful (and arguably necessary) to improve their results.

### Function as a structured label

Rather than treating the task as a collection of binary classification problems ("is a particular GO keyword associated with a particular protein?"), the GOstruct method trains a predictor to infer a full set of annotations directly ("what GO keywords are associated with a particular protein?") using the methodology of structured learning [1]. This is accomplished by learning a *compatibility function f *(**x**, **y**) that measures the level of association between a protein **x **and a vector of annotations **y**. Inference of anno-tations is then performed by finding the most compatible label with a given protein: **ŷ **= arg max**_y _***f *(**x**, **y**). An algorithm aimed at directly inferring complex labels such as GO annotations is called a *structured-output *method. Structured-output methods have been introduced to the field of machine learning fairly recently and span a number of discriminative and probabilistic approaches [26]. The most popular of these is the structured SVM, which shares many of the advantages of its binary counterpart [27]. Structured SVMs have been successfully applied to a variety of problems, including text categorization [27,28], prediction of disulfide-bond connectivity [29], and prediction of enzyme function [30], but are still not as widely used as binary SVMs due to their higher level of conceptual complexity and lack of easy to use software. In what follows we describe the extension of the GOstruct to multi-view classification.

## Methods

**Figure 1:**
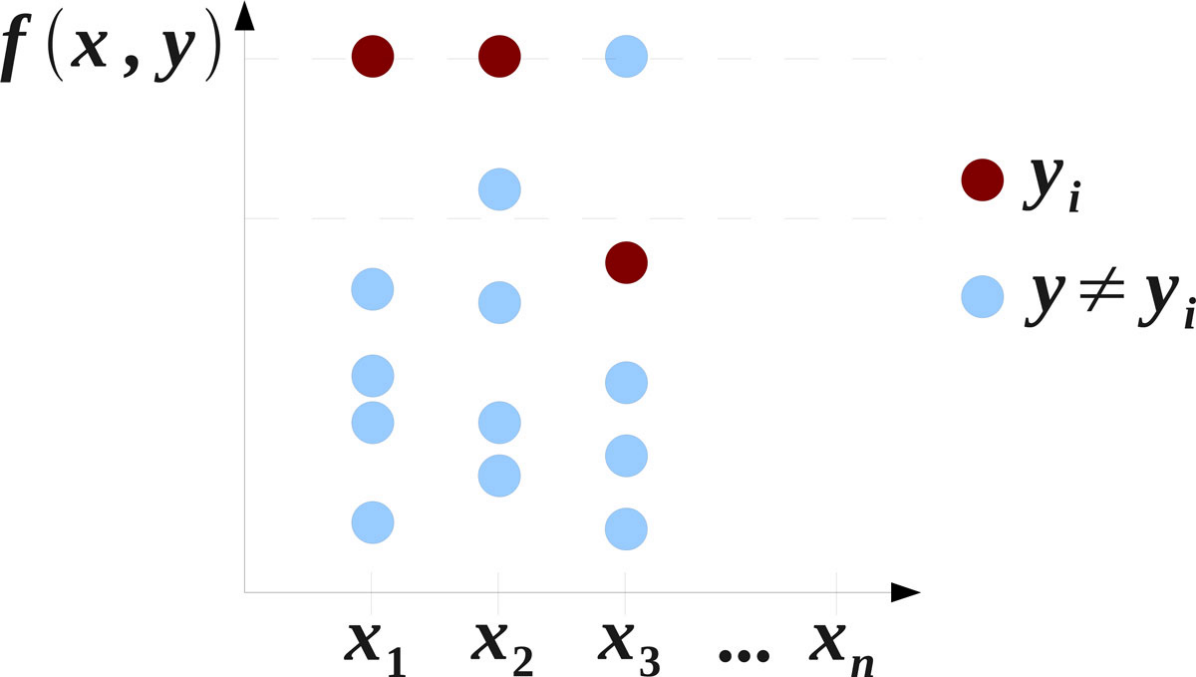
**Graphical representation of the training objective for structured-output methods**. Training examples are displayed along the horizontal axis. The structured SVM aims to maximize the margin between the compatibility values for the true label and all other labels, as depicted with the two dashed lines. Example **x**_1 _satisfies this. Example **x**_2_, while correctly classified, has a margin violation. Example **x**_3 _is misclassified. For demonstration purposes, we assume that the highest compatibility values for the three presented examples are all equal to each other.

Our labeled training data is provided as ${\left\{\left(x_{i},y_{i}\right)\right\}}_{i=1}^{n}\in {\left(X\;\times \;Y\right)}^{n}$, where X  is the space used to represent a protein, Y  is the label space, and *n *is the number of examples. Our goal is to construct an accurate mapping h:X→Y that minimizes the empirical loss $\sum_{i=1}^{n}\Delta \left(y_{i},h\left(x_{i}\right)\right)$ for a given loss function Δ. This mapping is computed using the compatibility function *f *via the arg max operator:

(1) $$h\left(x\right)=\underset{y\in Y}{\text{arg}\text{max}}f\left(x,y\right),$$

which selects the label **y **most compatible with the input **x**. The learning objective is then to ensure that the correct label **y***_i _*yields the highest compatibility score with **x***_i _*for every training example, as shown in Figure 1.

In this work, we utilize the structured SVM [27], which aims to maximize the separation between the compatibility value associated with the true label **y***_i _*and all other candidate labels for every training example **x***_i_*. The compatibility function for the structured SVM is linear in the joint input-output space defined by a feature map *ψ*: *f *(**x**, **y**) = **w***^T ^ψ*(**x**, **y**). The structured SVM can be formulated as the following quadratic optimization problem [27]:

(2) $${\text{min}}_{w,\xi }\frac{1}{2}{∥w∥}^{2}+\frac{C}{n}\sum_{i=1}^{n}\xi_{i}$$

(3) $$\begin{array}{c} s.t.\;w^{T}\left(\psi \left(x_{i},y_{i}\right)-\psi \left(x_{i},y\right)\right)\geq \Delta \left(y,y_{i}\right)-\xi_{i}\;\;\text{for}\;i=1,\ldots ,n;\;y\in Y\backslash \left\{y_{i}\right\} \end{array}$$

(4) $$\xi_{i}\geq 0\;\;\;\;\;\;\;\text{for}\;i=1,\ldots ,n,$$

where ξ*_i _*is the slack variable associated with margin violation for **x***_i_*, *C *is a user-specific parameter that controls the trade-off between two competing objectives: maximizing the margin through minimization of the norm of **w **and minimizing the amount of margin violation in the training data, as given by the sum of the slack variables. This is known as the margin-rescaling formulation of the structured SVM [27], because the margin with respect to which violations are measured is scaled according to how similar the true and the candidate labels are as measured by Δ(**y**, **y***_i_*). Here, we use the kernel *F*_1 _loss function [1]:

(5) $$\Delta_{ker}\left(y,\hat{y}\right)=1\frac{2K\left(y,\hat{y}\right)}{K\left(y,y\right)+K\left(\hat{y},\hat{y}\right)},$$

which reduces to the *F*_1_-loss [27] when using a linear kernel.

To make use of kernels, we solve the problem in Equations (2)-(4) in its dual formulation [27]. When dealing with structured-output problems, the kernels correspond to dot products in the joint input-output feature space defined by *ψ*, and the kernels are functions of both inputs and outputs: *K*((**x**_1_, **y**_1_), (**x**_2_, **y**_2_)) = *ψ*(**x**_1_, **y**_1_)*^T ^ψ*(**x**_2_, **y**_2_). In our experiments, we use a joint kernel that is the product of the input-space and the output-space kernels:

$$K\left(\left(x_{1},y_{1}\right),\left(x_{2},y_{2}\right)\right)=K_{X}\left(x_{1},x_{2}\right)K_{Y}\left(y_{1},y_{2}\right).$$

Our intuition is that two example-label pairs are similar if they are similar in both the input and the output spaces. The corresponding feature map *ψ *is given by all pair-wise combinations of the input-space and output-space features. Different sources of data are combined by adding kernels at the input-space level, and for the output space we use a linear kernel between label vectors. All kernels were normalized according to

$$K\left(z_{1},z_{2}\right)=\frac{K\left(z_{1},z_{2}\right)}{\sqrt{K\left(z_{1},z_{1}\right)K\left(z_{2},z_{2}\right)}}$$

to ensure consistent contribution across different feature spaces. Multiple sets of features were combined via unweighted kernel summation.

### Multi-view learning

**Figure 2:**
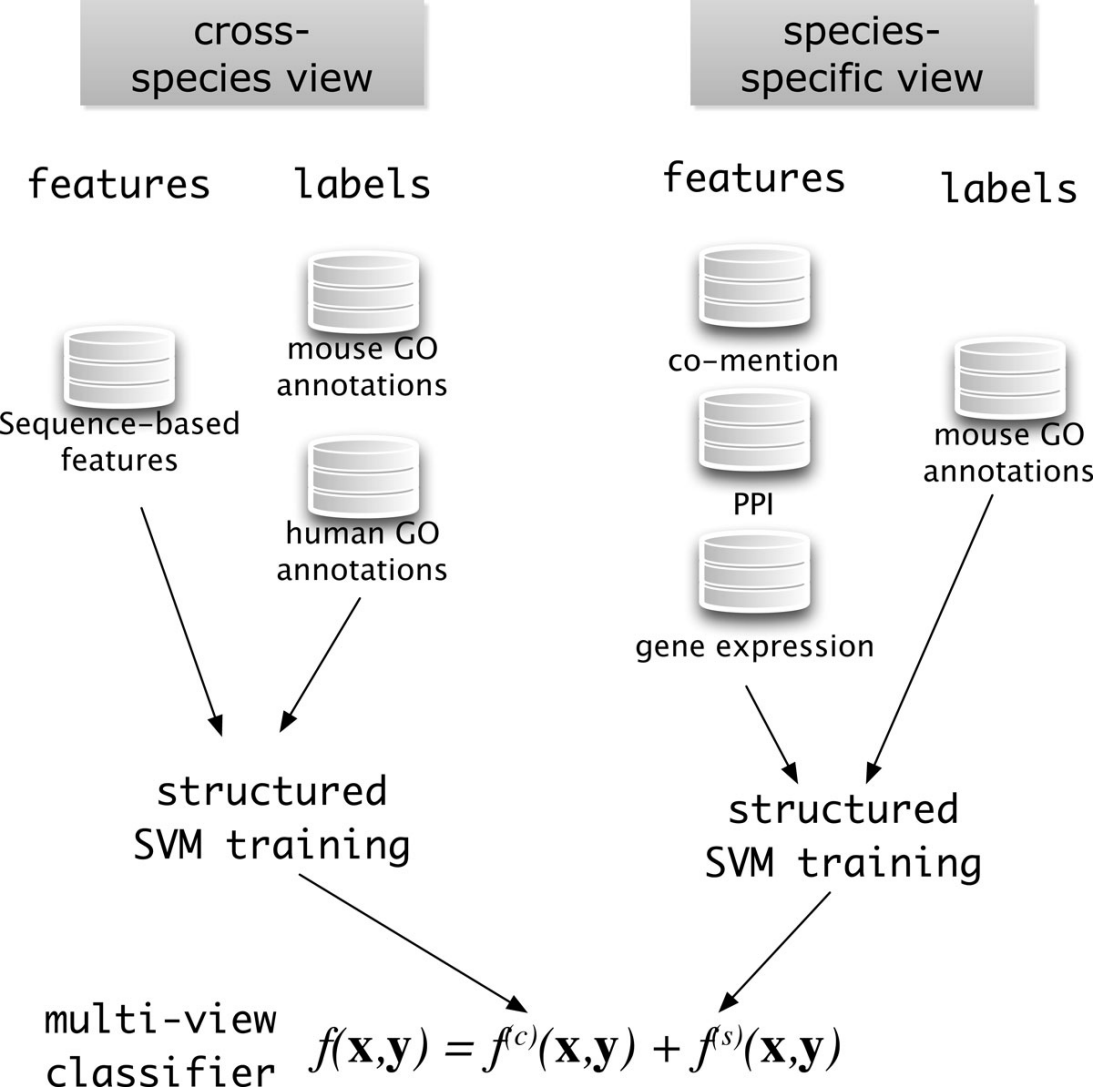
**The multi-view approach**. Data is separated into two views: a cross-species view that contains features computed from sequence, and a species-specific view that contains features computed from PPIs, gene expression and protein-GO term co-mention in mouse. A separate classifier is trained on the data from each view; the multi-view classifier uses the sum of the two compatibility functions.

The challenge in combining species-specific data such as gene expression and PPI data with sequence information in the structured SVM framework is that sequence is comparable across species whereas genomic data like gene expression and PPI data are not. To solve this problem we divide the data into two views: a *cross-species *view which will have a sequence-based kernel $K_{X}^{\left(C\right)}\left(x_{\text{1}},x_{\text{2}}\right)$ associated with it, and a *species-specific *view whose kernel $K_{X}^{\left(s\right)}\left(x_{\text{1}},x_{\text{2}}\right)$ will be computed from a collection of genomic data. Each view is trained in-dependently of the other using the margin-rescaling structured SVM formulation from Equations (2)-(4). As presented in Figure 2, the training leads to two compatibility functions: *f*^(*c*)^, which handles the cross-species view, and *f*^(*s*)^, which handles the species-specific view. Inference is then performed according to

(6) $$\overset{}{}h\left(x\right)\underset{y\in Y}{\text{ = arg}\text{max}}\left(f^{\left(c\right)}\left(x,y\right)+f^{\left(s\right)}\left(x,y\right)\right).$$

**Figure 3:**
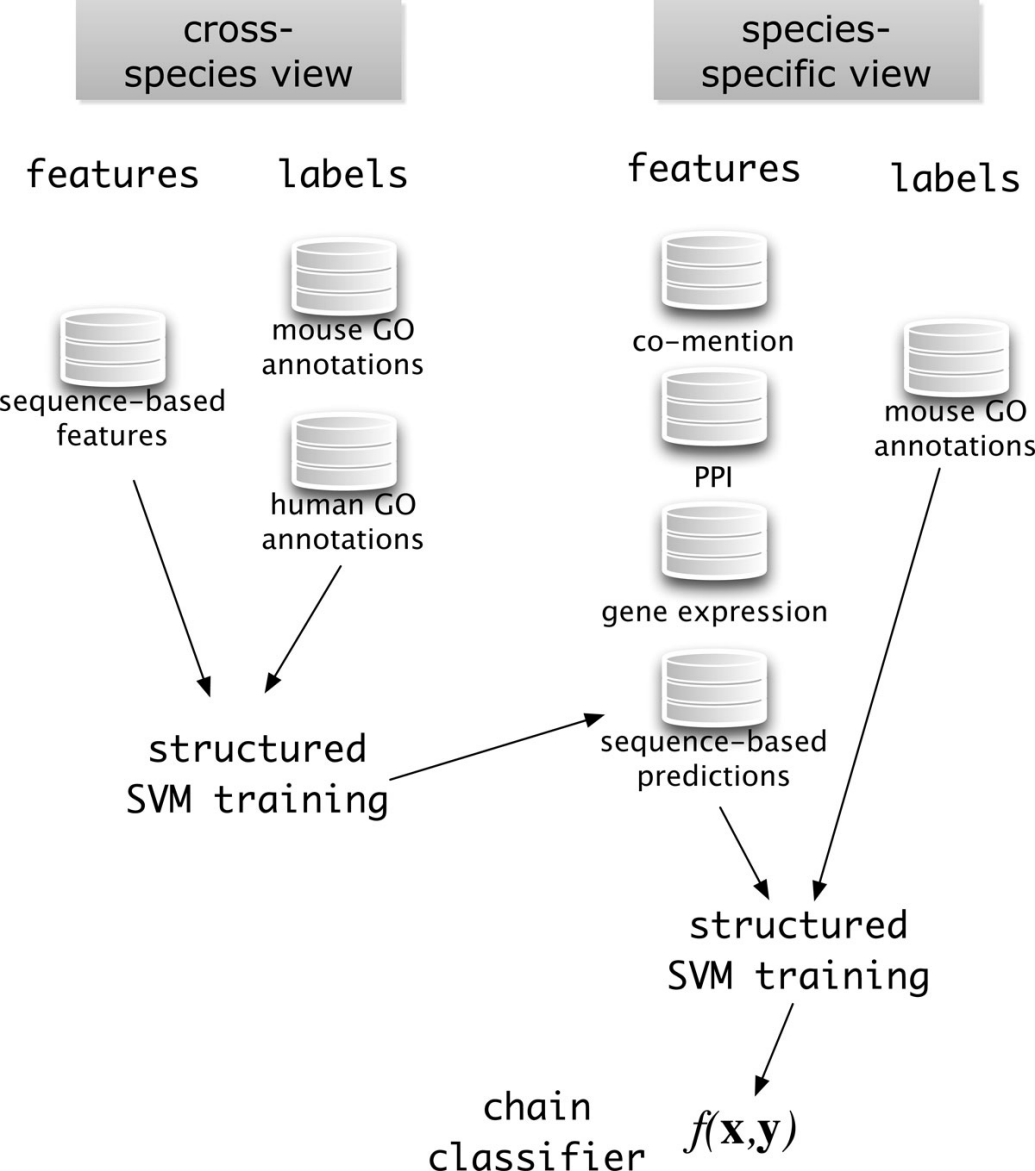
**The chain classifier approach**. Predictions from the cross-species view are provided as features to the species-specific view, along with other data.

In addition to the multi-view method outlined above, we investigate an approach we call the *chain *classifier. In this approach, the predictions made by the cross-species classifier are incorporated into the species-specific feature map by adding a feature for each GO term. In other words, arg max**_y _***f*^(*c*)^(**x***_i_*, **y**) becomes a set of features for the training of the species-specific classifier. The inference made by the species-specific classifier is then reported as the overall prediction. This approach is related to the method of Clark and Radivojac, which trains a neural network over GOtcha scores [31]. The chain approach, depicted in Figure 3, is an alternative way of learning from the training information available in the two views, and one of its advantages is that the user is not limited to structured SVMs for constructing the features from the cross-species view, and a simple BLAST nearest-neighbor approach can be used to produce predictions from the cross-species information instead of the structured SVM.

### Training and inference

To make inference via Equation (1) feasible we limited the output space Y  to the labels that occur in the training set only, arguing that this allows the classifier to focus on combinations of GO terms that are biologically relevant. We have found that it is possible to perform approximate inference using an efficient dynamic programming algorithm [32], but experiments have shown that performing inference that way leads to reduced accuracy, further supporting our choice to limit inference to combinations of GO terms observed in the data. We solve the SVM optimization problem in its dual formulation using the working set approach [27], which starts by optimizing the dual objective with no constraints. The algorithm then alternates between two steps: adding the most violated constraint to the working set, and solving the optimization problem with respect to the working set. The algorithm terminates when any constraint outside of the working set is violated by no more than any constraint in the working set.

### Measuring performance

Performance in hierarchical classification can be measured either at the level of individual GO terms, or by collectively comparing the discrepancy between the structured labels [1]. For ease of interpretability we choose to measure accuracy at the GO term level, and perform averaging across GO terms [21]. However, since GOstruct assigns a confidence to a set of predicted GO terms, we need to extract out of the compatibility function a confidence measure for *individual *GO terms. We compute a score *c_i_*(**x**) for GO term *i *on protein **x **according to:

(7) $$c_{i}\left(x\right)=\underset{y\in Y_{i}^{+}}{\text{max}}f\left(x,y\right)-\underset{y\in Y_{i}^{-}}{\text{max}}f\left(x,y\right),$$

where $Y_{i}^{+}=\left\{y\in Y|y_{i}=1\right\}$ is a subset of all labels that satisfy the hierarchical constraints and have the *i^th ^*variable set to 1. The subset $Y_{i}^{-}$ is defined in a similar fashion, except for the *i*^th ^variable being set to 0. This score measures the difference in compatibility values between the most compatible label that includes GO term *i *and the most compatible label that doesn't; higher values of this difference reflect stronger confidence that GO term *i *is associated with protein **x**. Given this score, we can generate ROC and precision-recall curves in the usual way; in our results we quantify performance using the area under the ROC curve (AUC), and the precision at a recall level of 20% (P20R).

### Evaluation procedure and model selection

Performance was evaluated using five-fold cross-validation on mouse proteins that have species-specific in-formation and valid annotations. Additional proteins, with cross-species features only, were obtained from the external species *H*. *sapiens*. In the interest of keeping the run times down, we randomly subsampled the external set down to 5000 proteins for molecular function and cellular component experiments and down to 3000 proteins for biological process experiments. Since sequence information was used, cross-validation folds were randomly selected such that no two proteins from different folds have more than 50% sequence identity. To select appropriate values for the SVM parameter *C*, we ran nested four-fold cross-validation on the training data. The value of $\frac{C}{n}=1$ yielded the highest accuracy on the validation set almost universally.

### Data

**Figure 4:**
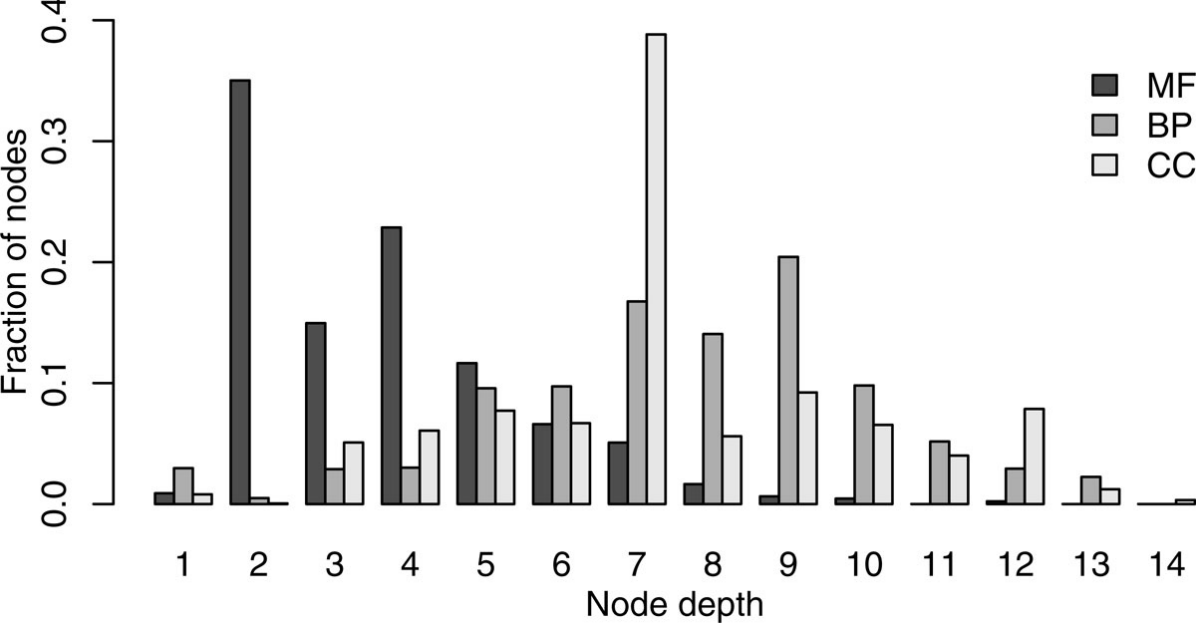
**The distribution of the GO term depth in the annotations provided by the dataset**. Term depth is computed as the length of the longest path to the root of the corresponding ontology.

As a target species we focus on *M*. *musculus*, and use *H*. *sapiens *as the external species that participates in the cross-species view. We choose the external species that is reasonably close to the target species and have a significant number of experimentally derived GO annotations. We obtained annotations from the Gene Ontology website (http://www.geneontology.org) and excluded annotations that were predicted through computational means to limit classifier assessment bias [33]. Table 1 and Figure 4 provide further information about each dataset.

**Table 1:** The number of proteins in mouse and human that participated in classifier training and testing, as well as the number of GO terms considered in each namespace.

Statistic	Namespace		
	MF	BP	CC
mouse proteins	3150	2633	2125
human proteins	5000	3000	5000
number of GO terms	310	1697	240

Namespace designations are as follows: MF - molecular function; BP - biological process; CC - cellular component.

### Cross-species data

We used features based on protein sequence to construct the cross-species view. Protein sequences for all species were retrieved from the UniProt database (http://uniprot.org). In the cases where a gene has multiple splice forms, the longest one was used. Sequence features were extracted as follows, and a linear kernel was used for the input space kernel for the cross-species view.

#### BLAST hits

We represented a protein in terms of its BLAST similarity scores against a database of annotated proteins [34]. We performed all-vs-all BLAST and the output was post-processed by excluding all hits with e-values above 50.0. The remaining e-values were divided by 50.0 to normalize them. Any values below 1e-10 after normalization were brought up to 1e-10. We then use the negative log of the resulting values as features.

#### Localization signals

Many biological processes are localized to particular cellular compartments. Information about protein localization can, therefore, be indicative of the function those proteins perform [10]. To take advantage of such information, we use the features computed by the WoLF PSORT algorithm [35].

#### Transmembrane protein predictions

A large fraction of proteins are embedded in one of the cellular membranes. Transmembrane proteins tend to be associated with certain functions, such as cell adhesion and transport of ions. Therefore information indicating whether a given protein is a transmembrane protein, and more specifically, how many transmembrane domains it has can also be indicative of protein function. For each protein, we estimated the number of transmembrane domains using the TMHMM program [36], and an indicator variable was associated with each number of transmembrane domains.

#### K-mer composition of the N and C termini

The N and C termini of a protein contain signals that are important for protein localization, binding and other protein functions [37]. Therefore we computed features that represent the 3-mer composition of 10 amino acid segments in the N and C termini of each protein.

#### Low complexity regions

Low-complexity regions in proteins are abundant, have an effect on protein function and are not typically captured by standard sequence comparison methods [38]. We scanned each protein with a sliding window of size 20, and a defined the low-complexity segment as the window that contains the smallest number of distinct amino acids. We used the amino acid composition of that segment as features.

### Species-specific data

We characterize functional similarity within a species using three sources of data: protein-protein interactions (PPI), gene expression, and protein-GO term co-mentions extracted from the biomedical literature.

#### Protein-protein interactions

We extracted *M*. *musculus *protein-protein interaction (PPI) data from version 8.3 of the STRING database [39]. A protein is represented by a vector of variables, where component *i *provides the STRING evidence score of an interaction between protein *i *and the given protein. Preliminary experiments indicate that the resulting linear kernel performs as well as the more sophisticated diffusion kernel.

#### Gene expression

Similarity of expression was measured using a linear kernel across a compendium of 14,696 microarray experiments provided by the authors of Platform for Interactive Learning by Genomics Results Mining (PILGRIM) [40]. Experiments using more sophisticated kernels will be provided elsewhere.

#### Protein-GO term co-mentions

If a protein and a gene ontology term are mentioned in close proximity in a paper, this can be evidence that the corresponding function is associated with the protein. A set of 11.7 million PubMed abstracts, all Medline abstracts on 9/8/2011 that had title and body text, were used to create a protein-GO term co-mention kernel. The abstracts were fed into a natural language processing pipeline based on the BioNLP UIMA resources (http://bionlp-uima.sourceforge.net/) which consists of the following steps: 1) splitting the abstracts into sentences 2) protein name tagging using the LingPipe named entity recognizer (http://alias-i.com/lingpipe) with the CRAFT model [41] 3) Gene Ontology term recognition via dictionary lookup and 4) extraction of protein-GO term co-occurrence at the abstract level. Protein names were mapped to mouse MGI IDs using MGI name dictionary lookup. Assuming only mouse references allowed us to avoid the full gene normalization problem [42] and fit in well with the other data sources of the species-specific classifier. The MGI ID-GO ID co-occurrence frequency data was used as features. In this data, each protein is characterized by a vector that provides the number of times it co-occurs with each GO term. In preliminary experiments we also explored the use of protein-protein co-occurrences, but found that they actually hurt performance.

Overall we extracted a total of 146,947,306 protein-GO term co-mentions. However, only 1,392,023 of those were unique - many GO-term protein pairs co-occur many times. An extreme example is interleukin 6, which is mentioned 426,031 times in conjunction with interleukin-6 receptor binding. Across the dataset, each protein co-occurred with a median of 50 molecular function, 117 biological process, and 42 cellular component GO-term mentions. Some basic statistics are presented in Table 2.

**Table 2:** Statistics of the co-mention data across GO namespaces.

Namespace	MF	BP	CC
Number of GO terms mentioned	3,611	6,684	1,296
Number of protein-GO term co-mentions	53,313,608	57,723,143	35,910,555
Number of unique co-mentions	376,498	768,876	246,649
Mean per protein	104	206	66
Std dev	131	250	70
Median	50	117	42
Range	1 - 1108	1 - 2034	1 - 531

We provide the number of GO terms mentioned, the number of unique co-mentions (number of protein-GO term pairs), and their total number, the average, median, standard deviation and range of the number of unique co-mentions per protein.

While it is clear from previous research that exact term matching is inadequate for good recall of Gene Ontology terms in text [43], it is also clear that accurately recognizing Gene Ontology terms is a challenging problem not only due to linguistic variation [24] but due to variability in term informativeness in the context of the GO itself [44]. Our conservative exact-match approach to recognizing GO terms is highly precise, and its low coverage is likely offset by the large document collection we have considered in this work. Our collection is orders of magnitude larger than previous collections (for instance, [25] uses 68,337 abstracts for training and the BioCreative data [22] consisted of 30,000 (full text) documents). Our use of direct protein mentions within a document to relate proteins to GO terms, and aggregated across the corpus as a whole, also differentiates this work from previous efforts that use externally provided protein-text links. In BioCreative, the test data consisted of protein-document pairs in the input and most systems considered only the information within the document(s) provided for a protein rather than *any *document in the collection that might mention the protein; [25] associates proteins to text via curated protein-document links in UniProt. This means our methods consider many more implied relationships than other methods.

## Results

### Comparing classification approaches

We trained cross-species, species-specific, multi-view and chain models and assessed their performance in prediction of mouse protein function using cross-validation as described above. The cross-species classifier uses only sequence information, the species-specific classifier uses PPI, gene expression, and protein-GO term co-mentions. The results in Table 3 demonstrate the advantage of the multi-view and chain approaches: these classifiers achieve the highest precision and AUC than either view by itself, and the multi-view approach is generally better than the chain method. The only exception is the biological process namespace, where the species-specific classifier achieves slightly better AUC (although worse P20R) than the multi-view and chain classifiers. This is the result of the relatively poor performance of the cross-species classifier in this case.

**Table 3:** Classifier performance in predicting GO terms in mouse, quantified by area under the ROC curve (AUC) and precision at 20% recall (P20R).

Namespace		*AUC*			*P20R*	
	MF	BP	CC	MF	BP	CC
Cross-species	0.90	0.67	0.81	0.52	0.16	0.42
Species-specific	0.86	0.83	0.86	0.42	0.29	0.46
Multi-view	0.91	0.81	0.88	0.57	0.30	0.58
Chain	0.89	0.82	0.87	0.51	0.28	0.52

The cross-species classifier uses only sequence data; the species-specific classifier uses a collection of genomic data--PPI, gene expression, and protein-GO term co-mention mined from the biomedical literature. The multi-view and chain classifiers are two approaches for integrating cross-species and species-specific data. The presented values are averages across all GO terms considered in a particular namespace. The results were obtained using five-fold cross-validation.

The cross-species SVM outperforms the species-specific SVM in molecular function, which is consistent with the literature demonstrating that molecular function annotations are the easiest to infer from sequence [33]. In the other two namespaces the species-specific SVM performs best, with the strongest contribution coming from the PPI data, as discussed below. This suggests that features that describe the functional network within a species are more predictive of biological process and cellular component than sequence-based features.

### Contribution from individual sources of data

To assess the contribution of each source of data to the prediction accuracy, we compared the performance of models trained on individual kernels. These results are presented in Table 4. Our first observation is that BLAST data accounts for the largest contribution to the predictive power of the cross-species SVM, although the additional sequence-based kernels provide an increase in performance. A further boost to performance in the cross-species view comes from the human sequence data, particularly in the molecular function namespace; this can be observed by comparing the "Sequence" entry in Table 4 to the "Cross-species" entry in Table 3.

**Table 4:** Classifier performance in predicting GO terms using individual sources of data and some of their combinations using only data from mouse.

Source		*AUC*			*P20R*	
	MF	BP	CC	MF	BP	CC
BLAST	0.77	0.61	0.69	0.40	0.13	0.25
Sequence	0.83	0.65	0.76	0.41	0.14	0.26

PPI	0.78	0.80	0.81	0.33	0.25	0.43
Protein-GO term co-mention	0.78	0.75	0.79	0.24	0.17	0.33
Expression	0.58	0.64	0.62	0.04	0.06	0.10
PPI + co-mention	0.85	0.82	0.85	0.43	0.29	0.45
PPI + co-mention + expression	0.86	0.83	0.86	0.42	0.29	0.46

BLAST refers to a classifier trained on BLAST scores only; the Sequence entry uses all the sequence-based features. In addition to classifiers trained on PPI, co-mention and expression individually, we also provide results using PPI and co-mention and the combination of all three.

In the species-specific view, the PPI kernel yields the highest accuracy, and outperforms all sequence-based predictors in biological function and cellular component namespaces, including the full cross-species SVM from Table 3. This suggests that functional network information, which is the basis for the "guilt by association" approach for function prediction is effective in those two namespaces (we note that the GOstruct framework was shown to outperform guilt by association methods in a comparison on the Mousefunc challenge data [1].) Furthermore, these features are complementary to the co-mention features, as demonstrated by the strong increase in performance over either kernel by itself when using the combination of the two. A classifier based solely on gene expression data did not fare well by itself. Nevertheless, inclusion of gene expression data provides a marginal increase in performance. Prediction of function from expression is challenging and others have observed poor performance using expression data alone [45]; we are currently exploring alternative representations that will improve its usefulness.

A manual analysis of incorrect predictions using literature features was performed to examine what information GOstruct used to make the prediction. Analysis of the top 25 false positives from the molecular function namespace can be found in Additional File 1 with the first few entries presented in Table 5. Three main conclusions can be drawn from the analysis. First, predictions made are more accurate than the evaluation allowed; our system identified biologically correct annotations that were not yet available in the gold standard. The gold standard used for evaluation was from Feb 2011. When evaluated against the contents of Swiss-Prot from April 2012, 16 out of the top 25 predictions are supported. Second, our NLP pipeline is able to extract pertinent information for function prediction. Even individual sentences can contain evidence of multiple GO annotations. For example, a sentence extracted by our pipeline from PMID:19414597, "LKB1, a master kinase that controls at least 13 downstream protein kinases including the AMP-activated protein kinase (AMPK), resides mainly in the nucleus.", describes both the function and the subcellular localization of the protein LKB1. Finally, even though the sentences extracted provide useful information, more sophisticated methods to extract information from them will need to be developed. Because we are using simple co-occurrence of protein and GO-terms, extracted associations are not always correct. For example, our pipeline associated peptidase activity with TIMP-2 on the basis of the following sentence: "The 72-kDa protease activity has been found to be inhibited by tissue inhibitor of metalloprotease-2 (TIMP-2), indicating that the protease is the matrix metalloprotease-2 (MMP-2)." Clearly, TIMP-2 does not actually have peptidase activity, but inhibits it. This incorrect association led to an incorrect GOstruct prediction. Such errors will be addressed in future work by incorporating the semantic role of the protein in regards to the described function. Overall, literature is a very informative feature for function predictions and continued work to develop more sophisticated methods for extracting protein-GO relations are required.

**Table 5:** The top 5 false positive predictions made by GOstruct.

Protein	GOstruct Prediction/Current Annotation (if dierent)	Best Supporting Sentence	Pubmed ID	GO term(s) in Supporting Sentence	Evidence Code
MGI:103293	GO:0016787 hydrolase activity	We recently demonstrated that human protein tyrosine phosphatase (PTP) L1, a large cytoplasmic phosphatase also known as PTP-BAS/PTPN13/PTP-1E, is a negative regulator of IGF-1R/IRS-1/Akt path-way in breast cancer cells.	19782949	GO:0004722	IEA
MGI:103305	GO:0016787 hydrolase activity/N/A	N/A	N/A	N/A	N/A
MGI:104597	GO:0016740 transferase activity/N/A	Using this assay system, chloramphenicol acetyltransferase activity directed by the cTNT promoter/upstream region was between two and three orders of magnitude higher in cardiac or skeletal muscle cells than in fibroblast cells, indicating that cis elements responsible for cell-specific expression reside in this region of the cTNT gene. Many Andersen syndrome cases have been associated with loss-of-function mutations in the inward rectifier K(+) channel Kir2.1 encoded by KCNJ2.	3047142	GO:0008811 GO:0016407	N/A
MGI:104744	GO:0022857 transmembrane transporter activity/GO:0005242 inward rectifier potassium channel activity		18690034	GO:0015267	IEA
MGI:104744	GO:0022892 substrate-specific transporter activity/GO:0005242 inward rectifier potassium channel activity	IRK1, but not GIRK1/GIRK4 channels, showed a marked specificity toward phosphates in the 4,5 head group positions.	10593888	GO:0015267	IEA

We present the best supporting sentence for the function of each protein, the document source, and the most recent known annotation along with the associated evidence code.

### Performance comparison on individual GO terms

For further analysis of performance we examined our classifiers in the context of individual GO terms. For each namespaces we wanted to see whether there are trends in performance as a function of the GO term depth, and whether there are certain categories that are particularly easy or difficult to predict. Overall, we observed a slight upward trend, with predictors achieving higher accuracy on terms deeper in the ontologies. This was most pronounced in the biological process namespace.

#### Molecular Function

**Figure 5:**
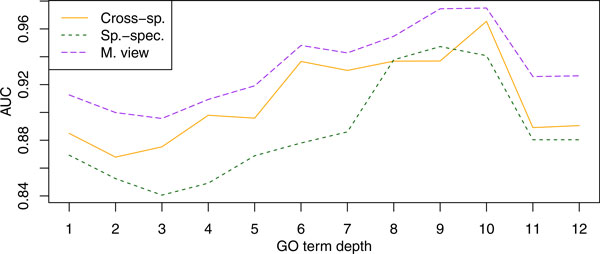
**Accuracy plotted against the GO term depth for the molecular function namespace**. Presented are average AUC values for three of the predictors in Table 3. Term depth is computed as the length of the longest path to the root of the ontology. The labels "Cross-sp.", "Sp.-Spec.", and "M. View" refer to the cross-species, species-specific and multi-view predictors, respectively.

Figure 5 presents the accuracy for molecular function GO terms. Among the more difficult to predict were several rather generic binding-related terms (GO:0019904 - "protein domain specific binding", GO:0019899 - "enzyme binding", and GO:0042802 - "identical protein binding", all with AUC values below 0.63 across all classifiers). A comparison of the cross-species classifier with the species-specific classifier shows that the cross-species classifier has better performance at predicting functions related to enzymatic activity (average AUC values of 0.93 and 0.89, respectively).

#### Biological Process

**Figure 6:**
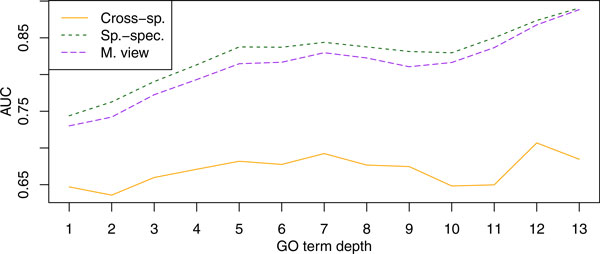
**Accuracy plotted against the GO term depth for the biological process namespace**. Presented are average AUC values for three of the predictors in Table 3. Term depth is computed as the length of the longest path to the root of the ontology. The labels "Cross-sp.", "Sp.-Spec.", and "M. View" are the same as above.

The results for the biological process namespace are presented in Figure 6. The striking feature of the results is that the species-specific view outperforms the cross-species classifier on almost every GO term. The difference in performance was largest in the more specific terms, which corresponds to the right part of the plot. Among the most difficult terms, on which all three predictors performed poorly, were GO:0019725 - "cellular homeostasis", GO:0040007 - "growth", and GO:0065003 - "macromolecular complex assembly"; the corresponding AUC values were below 0.67.

#### Cellular Component

**Figure 7:**
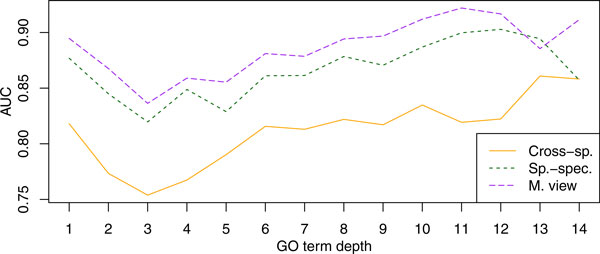
**Accuracy plotted against the GO term depth for the cellular component namespace**. Presented are average AUC values for three of the predictors in Table 3. Term depth is computed as the length of the longest path to the root of the ontology. The labels "Cross-sp.", "Sp.-Spec.", and "M. View" are the same as above.

Figure 7 presents the cellular component results. Similar to biological process, the species-specific classifier outperformed the cross-species one on nearly all GO terms; the only terms on which the cross-species does better are very general (e.g., "extracellular region"). The most difficult terms in this namespace were GO:0000267 - "cell fraction" and GO:0005829 - "cytosol" with the corresponding AUC values being below 0.73 across all predictors.

## Conclusions

This paper presented a multi-view extension to the GOstruct structured output protein function prediction framework. We demonstrated the framework's capability to combine multiple heterogeneous sources of data--annotated proteins from multiple species, and species-specific data that includes PPIs, gene expression, and information mined from the biomedical literature--each providing an increase in performance. The empirical results suggest that sequence-based features are more informative of a protein's molecular function, while functional association features from PPI and text mining data provide a stronger contribution for the prediction of biological process and cellular component annotations. Gene expression provided only a marginal increase in performance and we speculate that more sophisticated kernels are needed to extract more meaningful features. Future work includes the design of these kernels as well as framework extensions to make it more scalable to a higher number of species and larger datasets.

## Competing interests

The authors declare that they have no competing interests.

## Authors' contributions

AS and KG collected protein sequence, gene expression and protein-protein interaction data. CF collected protein-GOterm co-mention data. AS implemented the GOstruct framework, performed the baseline set of experiments and wrote the first versions of the manuscript. AS and KG performed the experiments involving gene expression data. KV planned and directed the NLP experiments, performed data analysis, and wrote or integrated text for the sections pertaining to NLP experiments. KV and CF performed all the experiments and analysis of results pertaining to the NLP portion of the manuscript. ABH supervised all aspects of the work. All authors read and approved the final manuscript.

## Supplementary Material

Additional file 1 **Analysis of the top 25 false positive predictions made by GOstruct**. We present the best supporting sentence for the function of each protein, the document source, and the most recent known annotation along with the associated evidence code. Click here for file

## Declarations

This article has been published as part of *BMC Bioinformatics *Volume 14 Supplement 3, 2013: Proceedings of Automated Function Prediction SIG 2011 featuring the CAFA Challenge: Critical Assessment of Function Annotations. The full contents of the supplement are available online at http://www.biomedcentral.com/bmcbioinformatics/supplements/14/S3.
