# Supplementary material for: Combining heterogeneous data sources for accurate functional annotation of proteins
Source: BMC Bioinformatics. 2013 Feb 28;14(Suppl 3):S10. doi: 10.1186/1471-2105-14-S3-S10 (PMC3584846; doi:10.1186/1471-2105-14-S3-S10)
Supplement: Additional file 1 — Analysis of the top 25 false positive predictions made by GOstruct. We present the best supporting sentence for the function of each protein, the document source, and the most recent known annotation along with the associated evidence code. [file 1471-2105-14-S3-S10-S1.PDF]

Protein	GOstruct Prediction / Current Annotation (if different)	Best Supporting Sentence	Pubmed ID	GO term(s) in Supporting Sentence	Evidence Code
MGI:103293	GO:0016787 hydrolase activity	We recently demonstrated that human protein tyrosine phosphatase (PTP) L1, a large cytoplasmic phosphatase also known as PTP-BAS/PTPN13/PTP-1E, is a negative regulator of IGF-1R/IRS-1/Akt pathway in breast cancer cells.	19782949	GO:0004722	IEA
MGI:103305	GO:0016787 hydrolase activity / N/A	N/A	N/A	N/A	N/A
MGI:104597	GO:0016740 transferase activity / N/A	Using this assay system, chloramphenicol acetyltransferase activity directed by the cTNT promoter/upstream region was between two and three orders of magnitude higher in cardiac or skeletal muscle cells than in fibroblast cells, indicating that cis elements responsible for cell-specific expression reside in this region of the cTNT gene.	3047142	GO:0008811 GO:0016407	N/A
MGI:104744	GO:0022857 transmembrane transporter activity / GO:0005242 inward rectifier potassium channel activity	Many Andersen syndrome cases have been associated with loss-of-function mutations in the inward rectifier K(+) channel Kir2.1 encoded by KCNJ2.	18690034	GO:0015267	IEA
MGI:104744	GO:0022892 substrate-specific transporter activity / GO:0005242 inward rectifier potassium channel activity	IRK1, but not GIRK1/GIRK4 channels, showed a marked specificity toward phosphates in the 4,5 head group positions.	10593888	GO:0015267	IEA
MGI:105926	GO:0005515 protein binding	Based on our results together with previous work showing that Rin1 interacts with signal transducing adapter molecule to facilitate the degradation of EGFR, we hypothesize that the selective association of Rab5A and Rin1 contributes to the dominance of Rab5A in EGFR trafficking	19723633	GO:0005488	IPI
MGI:105938	GO:0005515 protein binding / GO:0030742 GTP-dependent protein binding	To validate this method, the binding of EEA-1 was confirmed and several novel Rab5-binding proteins were also identified by 2-dimensional electrophoresis and liquid chromatography-mass spectrometry/mass spectrometry (LC-MS/MS).	19526728	GO:0017091 GO:0005488	IEA
MGI:107548	GO:0005515 protein binding / N/A	In vitro binding assays revealed that TRAF5 associates with the cytoplasmic tail of CD40, but not with the cytoplasmic tail of tumor receptor factor receptor type 2, which associates with TRAF2.	8790348	GO:0005515 GO:0003818	N/A
MGI:1316660	GO:0005515 protein binding / N/A	Members of the voltage-gated calcium channel y subunit gene family (Cacng), have been rapidly discovered since the discovery of the identification of the mouse gamma2 gene (Cacng2) and its association with the stargazer mutant mouse line.	15000525	GO:0015267 GO:0005262	N/A
MGI:1341870	GO:0016301 kinase activity	LKB1, a master kinase that controls at least 13 downstream protein kinases including the AMP-activated protein kinase (AMPK), resides mainly in the nucleus.	19414597	GO:0050405	IEA
MGI:1341870	GO:0016740 transferase activity	LKB1 can phosphorylate the Thr174 of BRSK2, increasing its activity >50-fold.	16870137	GO:0016310	IEA
MGI:1341870	GO:0016772 transferring phoshorus-containing groups	LKB1 tumour suppressor protein kinase phosphorylates and activates protein kinases belonging to the AMP activated kinase (AMPK) subfamily	15733851	GO:0004674	IEA

Protein	GOstruct Prediction / Current Annotation (if different)	Best Supporting Sentence	Pubmed ID	GO term(s) in Supporting Sentence	Evidence Code
MGI:1343087	GO:0016740 transferase activity	PKCzeta thus functions as an adaptor, associating with a staurosporine-insensitive PDK2 enzyme that catalyzes the phosphorylation of S472 of PKBgamma.	12162751	GO:0004697 GO:0004740	IEA
MGI:1343087	GO:0016772 transferring phoshorus-containing groups / GO:0004740 pyruvate dehydrogenase kinase activity	PKCzeta thus functions as an adaptor, associating with a staurosporine-insensitive PDK2 enzyme that catalyzes the phosphorylation of S472 of PKBgamma.	12162751	GO:0004697 GO:0004740	IEA
MGI:1926334	GO:0016787 hydrolase activity / GO:0004722 protein serine/threonine phosphatase activity	The protein B-50 is dephosphorylated in rat cortical synaptic plasma membranes (SPM) by protein phosphatase type 1 and 2A (PP-1 and PP-2A)-like activities.	1319470	GO:0004722	IDA
MGI:1926334	GO:0016788 hydrolase activity, acting on ester bonds / GO:0004722 protein serine/threonine phosphatase activity	The protein B-50 is dephosphorylated in rat cortical synaptic plasma membranes (SPM) by protein phosphatase type 1 and 2A (PP-1 and PP-2A)-like activities.	1319470	GO:0004722	IDA
MGI:2140494	GO:0016787 hydrolase activity / N/A	Nuclear inhibitor of protein phosphatase-1 (NIPP1; 351 residues) is a nuclear RNA-binding protein that also contains in its central domain two contiguous sites of interaction with the catalytic subunit of protein phosphatase-1 (PP1(C)).	11104670	GO:0016791 GO:0003723	N/A
MGI:2140494	GO:0016788 hydrolase activity, acting on ester bonds / N/A	Nuclear inhibitor of protein phosphatase-1 (NIPP1; 351 residues) is a nuclear RNA-binding protein that also contains in its central domain two contiguous sites of interaction with the catalytic subunit of protein phosphatase-1 (PP1(C)).	11104670	GO:0016791 GO:0003723	N/A
MGI:2180854	GO:0005515 protein binding / N/A	We report here that RFXAP, a subunit of the DNA-binding RFX complex, also binds BRG1 and therefore provides a mechanism by which MHC class II gene chromatin can be remodeled in the absence of CIITA.	15781111	GO:0005515 GO:0003677 GO:0017091	N/A
MGI:2385847	GO:0005515 protein binding	In contrast with other MOs, this conformational switch is coupled with the opening of a channel to the active site, suggestive of a protein substrate.	16275925	GO:0005515 GO:0015267	IPI
MGI:96785	GO:0005515 protein binding	Here, using the yeast one-hybrid system and electrophoretic mobility shift assay, we report that Lhx2, a LIM-homeodomain protein, binds to the homeodomain site in the mouse M71 OR promoter region.	15173589	GO:0005515 GO:0017091	IPI
MGI:97531	GO:0005515 protein binding	Many proteins bind to the activated platelet derived growth factor receptor (PDGF-R) either directly or by means of adapter molecules.	8619809	GO:0005515	IPI
MGI:97809	GO:0016787 hydrolase activity	We conclude that VE-PTP is a Tie-2 specific phosphatase expressed in ECs, and VE-PTP phosphatase activity serves to specifically modulate Angiopoietin/Tie-2 function.	10557082	GO:0004722 GO:0004725 GO:0016791	IEA
MGI:98753	GO:0008233 peptidase activity / N/A	The 72-kDa protease activity has been found to be inhibited by tissue inhibitor of metalloprotease-2 (TIMP-2), indicating that the protease is the matrix metalloprotease-2 (MMP-2).	12102173	GO:0008233 GO:0004222	N/A

Protein	GOstruct Prediction / Current Annotation (if different)	Best Supporting Sentence	Pubmed ID	GO term(s) in Supporting Sentence	Evidence Code
MGI:98753	GO:0016787 hydrolase activity / N/A	In the comparison of normal and cloned samples, a total of 41 spots were identified as differentially expressed proteins, of which 25 spots were up-regulated proteins such as TIMP-2, glutamate-ammonia, and esterase 10, while 16 spots were down-regulated proteins such as PBEF and annexin A1.	20684987	GO:0004091	N/A

Analysis of the top 25 false positive predictions made by GOstruct. We present the best supporting sentence for the function of each protein, the document source, and the most recent known annotation along with the associated evidence code.
